# The Impact of Preoperative Breast MRI on Surgical Margin Status in Breast Cancer Patients Recalled at Biennial Screening Mammography: An Observational Cohort Study

**DOI:** 10.1245/s10434-021-09868-1

**Published:** 2021-04-01

**Authors:** Jessie J. J. Gommers, Lucien E. M. Duijm, Peter Bult, Luc J. A. Strobbe, Toon P. Kuipers, Marianne J. H. Hooijen, Ritse M. Mann, Adri C. Voogd

**Affiliations:** 1 Department of Medical Imaging, Radboud University Medical Center, Nijmegen, The Netherlands; 2 Department of Radiology, Canisius Wilhelmina Hospital, Nijmegen, The Netherlands; 3 Department of Pathology, Radboud University Medical Center, Nijmegen, The Netherlands; 4 Department of Surgical Oncology, Canisius Wilhelmina Hospital, Nijmegen, The Netherlands; 5 Department of Radiology, Bernhoven Hospital, Uden, The Netherlands; 6 Department of Radiology, St Anna Hospital, Geldrop, The Netherlands; 7 Department of Radiology, Netherlands Cancer Institute, Amsterdam, The Netherlands; 8 Department of Epidemiology, Maastricht University, Maastricht, The Netherlands

## Abstract

**Background:**

This study aimed to examine the association between preoperative magnetic resonance imaging (MRI) and surgical margin involvement, as well as to determine the factors associated with positive resection margins in screen-detected breast cancer patients undergoing breast-conserving surgery (BCS).

**Methods:**

Breast cancer patients eligible for BCS and diagnosed after biennial screening mammography in the south of The Netherlands (2008–2017) were retrospectively included. Missing values were imputed and multivariable regression analyses were performed to analyze whether preoperative MRI was related to margin involvement after BCS, as well as to examine what factors were associated with positive resection margins, defined as more than focally (>4 mm) involved.

**Results:**

Overall, 2483 patients with invasive breast cancer were enrolled, of whom 123 (5.0%) had more than focally involved resection margins. In multivariable regression analyses, preoperative MRI was associated with a reduced risk of positive resection margins after BCS (adjusted odds ratio [OR] 0.56, 95% confidence interval [CI] 0.33–0.96). Lobular histology (adjusted OR 2.86, 95% CI 1.68–4.87), large tumor size (per millimeter increase, adjusted OR 1.05, 95% CI 1.03–1.07), high (>75%) mammographic density (adjusted OR 3.61, 95% CI 1.07–12.12), and the presence of microcalcifications (adjusted OR 4.45, 95% CI 2.69–7.37) and architectural distortions (adjusted OR 1.85, 95% CI 1.01–3.40) were independently associated with positive resection margins after BCS.

**Conclusions:**

Preoperative MRI was associated with lower risk of positive resection margins in patients with invasive breast cancer eligible for BCS using multivariable analysis. Furthermore, specific mammographic characteristics and tumor characteristics were independently associated with positive resection margins after BCS.

Since the 1980s, breast-conserving surgery (BCS) has gradually replaced mastectomy as the standard treatment for early breast cancer.^1^ BCS requires complete removal of the tumor, as positive resection margins have shown to be associated with an increased risk of local recurrence.^2^,^3^ In case of tumor-positive resection margins after BCS, a re-excision or mastectomy is performed. Reoperations are associated with physical and emotional burden for the patient, worse cosmetic results, and higher healthcare costs.^4^ Consequently, the re-excision rate is a national breast treatment quality indicator.^5^

Preoperative assessment of the extent of disease is crucial for surgical planning. To assess tumor size and location, clinical examination, mammography, and ultrasound are usually performed. However, measurement of tumor size by clinical examination and conventional imaging correlates poorly with histopathologic tumor size.^6^ Breast magnetic resonance imaging (MRI) has been shown to be more accurate than mammography or ultrasound to evaluate tumor size, multifocality, and the presence of contralateral breast cancer.^7^–^9^ As a result, breast MRI is increasingly being used in the preoperative evaluation of breast cancer.^10^

Despite its increased use, the clinical value of preoperative breast MRI in patients with breast cancer undergoing BCS remains a topic of debate. The fact that breast MRI detects additional disease, not seen with conventional imaging, has created the impression that preoperative MRI improves surgical planning and the likelihood of complete tumor excision.^7^,^11^ However, meta-analyses have shown that preoperative MRI might lead to higher mastectomy rates without reducing re-excision rates after BCS.^12^,^13^ Since 2012, the use of preoperative MRI in The Netherlands has been advised in patients with invasive lobular breast cancer and in case of discrepancies between physical examination, mammography, and/or ultrasound.^14^

Most studies on the potential benefits of preoperative MRI have included patients with clinically detected breast cancer. To our knowledge, data on the value of preoperative breast MRI in screen-detected cancer is lacking. Cancers detected in a screening program are usually non-palpable and smaller compared with clinically detected breast cancers. Exact localization of these screen-detected cancers is very important to be able to obtain clear resection margins. We used data from women who were recalled at biennial screening mammography in the south of The Netherlands to assess whether preoperative MRI reduces the risk of positive resection margins in patients with screen-detected breast cancer undergoing BCS. Second, we aimed to determine the factors associated with the risk of positive resection margins in patients with screen-detected breast cancer undergoing BCS.

## Methods

### Study Population

**Fig. 1:**
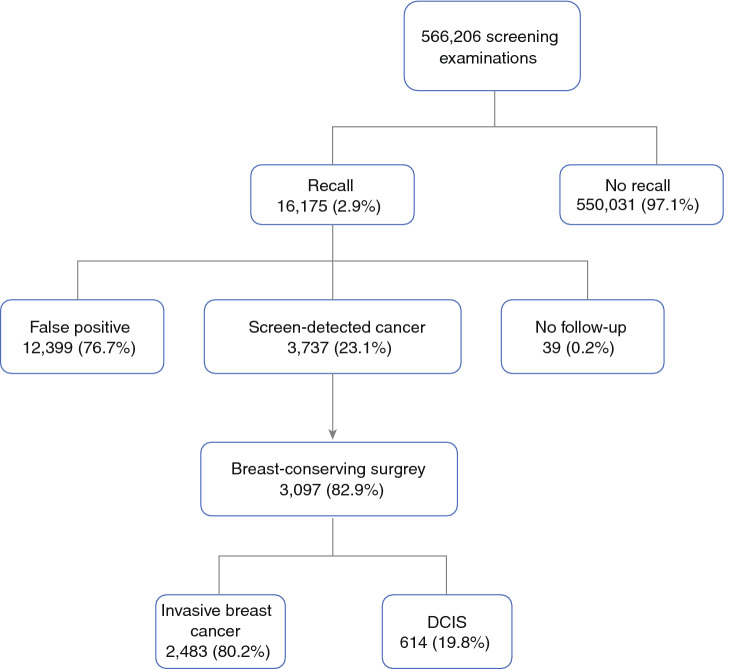
Breast cancer screening examinations and subsequent outcomes, from 2008 to 2017. *DCIS* ductal carcinoma in situ

We retrospectively analyzed all recalled women who received screening mammography at one of the four screening units in the south of The Netherlands between 1 January 2008 and 31 December 2017. Before being screened, women were offered the possibility to opt out of the use of their data for quality assurance and scientific purposes. Two women used this option and were therefore excluded from analysis. A total of 566,206 mammography screening examinations (61,635 initial screening examinations and 504,571 subsequent screening examinations) were included in this study. Figure 1 shows a flowchart of the study population. In total, 3737 women were diagnosed with a screen-detected breast cancer, of whom 3097 (82.9%) underwent BCS and were available for analysis on resection margins. According to the Dutch Central Committee on Research involving Human Subjects, ethical approval was not necessary.^15^ Our study was conducted in accordance with the Declaration of Helsinki.

### Screening Procedure

In The Netherlands, women aged 50–75 years are invited to attend biennial screening mammography. Details of the nationwide screening program have been described elsewhere.^16^–^18^ In summary, screening mammograms were obtained by screening mammography radiographers and independently read by two certified screening radiologists. The screening radiologists classified the mammograms according to the Breast Imaging Reporting and Data System (BI-RADS).^19^,^20^ Women with normal (BI-RADS 1) or benign (BI-RADS 2) findings were invited to re-attend the biennial screening program (except for women who were in the last screening round). Women with mammographic abnormalities (BI-RADS 0, incomplete; 4, suspicious of malignancy; 5, highly suggestive of malignancy) were recalled for additional work-up at a hospital. BI-RADS 3 category is not used in the screening program as short-term follow-up is not available within our screening program. In case of a discordant reading between two screening radiologists (before 2015), one classifying the mammogram as BI-RADS 1 or 2 (no recall) and the other as BI-RADS 0, 4 or 5 (recall), the woman was recalled without a consensus meeting. From 2015 on, discordant readings were read by a third radiologist. The mammographic abnormalities of women who were recalled were categorized into one of the following categories: suspicious mass, suspicious microcalcifications, suspicious mass with microcalcifications, architectural distortion, asymmetry, or other suspicious abnormality. Breast density was classified according to BI-RADS.^19^

### Diagnostic Work-Up After Recall

A total of thirty hospitals were involved in the diagnostic work-up. At the hospitals, women underwent breast mammography, as well as physical examination. The clinical radiologist classified the mammogram according to the BI-RADS score.^19^,^20^ BI-RADS 4 or 5 lesions were routinely biopsied and BI-RADS 3 lesions were either biopsied or followed-up, as decided by the multidisciplinary breast team. Women with BI-RADS 1 or 2 lesions were referred back to the screening program. Which tests to use for the diagnostic work-up was at the discretion of the breast radiologist and could include mammography, breast tomosynthesis and/or ultrasound in combination with tissue sampling of suspicious lesions, according to Dutch and European guidelines.^14^,^21^ Furthermore, patients could be discussed in multidisciplinary breast team meetings, in which the necessity to perform breast MRI was discussed, while considering the Dutch guidelines.^14^ Indications for preoperative MRI included dense breast tissue as well as invasive lobular breast cancer, and breast MRIs were performed to improve delineation of tumor size, to detect ipsilateral (multifocal or multicentric) disease, to exclude or confirm contralateral disease, and to help the surgeon decide for BCS or mastectomy. Breast MRIs were performed in different hospitals, using 1.5T or 3.0T MRI machines from different vendors. In each hospital, dynamic contrast-enhanced MRI was performed according to local protocol, adhering to the quality criteria suggested by the European Society of Breast Imaging (EUSOBI).^22^

### Follow-Up of Recalled Women

For all recalled women, radiology, biopsy, and surgery reports were obtained and collected in Microsoft Excel (Microsoft Corporation, Redmond, WA, USA). If a woman was recalled for more than one lesion in a breast or for bilateral lesions during the same screening round, the lesion with the highest suspicion at mammography was considered as the index lesion for recall.

Breast cancers were divided into ductal carcinoma in situ (DCIS) and invasive cancers. Lobular carcinoma in situ (LCIS) was classified as a lesion not needing treatment, except pleomorphic LCIS, which was treated as DCIS. Cancers were classified according to the Union for International Cancer Control (UICC) TNM classification. Until 2009, the UICC 6th edition was used,^23^ and from 2010 until 2016 and from 2017 onwards, the UICC 7th^24^ and 8th editions^25^ were used, respectively. Lymph nodes were considered negative (N−) if they contained no tumor or only isolated cells (≤0.2 mm), and were classified as positive (N+) if they contained micrometastases (>0.2–2 mm) or macrometastases (>2 mm). If ≥10% of the cancer cells showed nuclear staining, estrogen and progesterone status were considered positive.^14^ Human epidermal growth factor receptor 2 (HER2) status was classified as positive in cases of HER2 immunohistochemistry (IHC) 3+ or HER2 IHC 2+ and amplified within in situ hybridization.^14^ Surgical margin status was registered as negative, focally positive (≤4 mm involved margin), or more than focally positive (>4 mm involved margin), according to the Dutch guideline for breast cancer.^14^ A more than focally involved margin was an indication for re-excision or mastectomy. In this study, specimens with more than focally involved margins were considered as tumor-positive margins and specimens with focally positive margins were considered as tumor-negative margins.

### Statistical Analysis

Breast cancer patients who underwent BCS as primary treatment were eligible for inclusion. The study population was divided into a no MRI and an MRI group according to the preoperative use of breast MRI. Differences in patient and tumor characteristics between the two groups were tested using the Mann–Whitney U test for continuous variables and the Chi-square test for categorical variables. Univariable and multivariable binary logistic regression analyses were performed to determine the association between preoperative MRI and the presence of positive resection margins (>4 mm involved margin) after BCS. The multivariable method was performed by the enter method and included the variable preoperative MRI, as well as all variables that were associated with positive resection margins in univariable analysis (*p*-value <0.10). Missing values on covariates were imputed using multiple imputation (MI). If missing values showed a monotone pattern, the monotone MI method was used, but if the missing pattern was not monotone, the fully conditional specification was used.

Statistical analyses were performed using IBM SPSS Statistics version 25.0 (IBM SPSS Statistics for Windows, IBM Corporation, Armonk, NY, USA). Statistical tests were two-sided and *p*-values <0.05 were regarded as statistically significant.

## Results

### Patient and Tumor Characteristics

Overall, 3737 women were diagnosed with a screen-detected breast cancer, of whom 3097 (82.9%) underwent BCS, 588 (15.7%) underwent mastectomy, and 52 (1.4%) underwent no surgery or their surgery was unknown. The percentage of women undergoing preoperative breast MRI was 15.8% in the BCS group and 46.4% in the mastectomy group.

A total of 3097 patients underwent BCS and were thus eligible for inclusion. Invasive cancer was diagnosed in 2483 patients, of whom 454 (18.3%) had received preoperative MRI. Pure DCIS was diagnosed in 614 women, of whom 35 (5.7%) had received preoperative MRI. Given the small number of women with DCIS who underwent preoperative MRI, we limited our analyses to women with invasive breast cancer.

Patient and tumor characteristics for women with invasive breast cancer are summarized in Table 1. Compared with women without preoperative MRI, women with preoperative MRI were generally younger (*p* < 0.001) and were found to have denser breasts (*p* < 0.001). Women with preoperative MRI had a higher proportion of masses with microcalcifications and architectural distortions on screening mammography, when compared with women without preoperative MRI (*p* < 0.001). Tumor histology also differed between women with and without preoperative MRI, with a higher proportion of invasive lobular cancers and less invasive ductal cancers among women who underwent preoperative MRI (*p* < 0.001). Furthermore, the tumors of women with preoperative MRI were generally larger (*p* < 0.001), more often lymph node-positive (*p* < 0.001), more frequently classified as Bloom and Richardson grade II (*p* < 0.001), and more often HER2 receptor-positive (*p* = 0.017) compared with the tumors of women without preoperative MRI.

**Table 1:** Patient and tumor characteristics of the 2483 patients with invasive breast cancer diagnosed after recall at screening mammography

	Total [*N* = 2483]	Preoperative MRI [*n* = 454]	No preoperative MRI [*n* = 2029]	*p*-value
Age, years				<0.001^a,^*
<60	868 (35.0)	205 (45.2)	663 (32.7)	
60–70	1176 (47.4)	199 (43.8)	977 (48.2)	
>70	439 (17.7)	50 (11.0)	389 (19.2)	
Breast density at screening mammogram, %				<0.001^a,^*
0–25	690 (34.6)	73 (21.1)	617 (37.4)	
25–50	925 (46.4)	161 (46.5)	764 (46.4)	
50–75	347 (17.4)	99 (28.6)	248 (15.0)	
75–100	32 (1.6)	13 (3.8)	19 (1.2)	
Unknown	489	108	381	
Mammographic abnormality				<0.001^a,^*
Mass	1810 (72.9)	288 (63.4)	1522 (75.0)	
Microcalcifications	210 (8.5)	38 (8.4)	172 (8.5)	
Mass with microcalcifications	199 (8.0)	51 (11.2)	148 (7.3)	
Asymmetry	78 (3.1)	17 (3.7)	61 (3.0)	
Architectural distortion	164 (6.6)	51 (11.2)	113 (5.6)	
Other	22 (0.9)	9 (2.0)	13 (0.6)	
Tumor histology				<0.001^a,^*
Ductal	1992 (80.2)	260 (57.3)	1732 (85.4)	
Lobular	266 (10.7)	163 (35.9)	103 (5.1)	
Mixed ductal-lobular	78 (3.1)	19 (4.2)	59 (2.9)	
Other	147 (5.9)	12 (2.6)	135 (6.7)	
Tumor size, mm^c^	12 (8–17)	16 (12–24)	11 (8–15)	<0.001^b,^*
Lymph node status				<0.001^a,^*
N+	463 (19.1)	137 (30.5)	326 (16.5)	
N−	1967 (80.9)	312 (69.5)	1655 (83.5)	
Unknown	53	5	48	
Bloom and Richardson grade				<0.001^a,^*
I	1145 (46.4)	165 (36.4)	980 (48.6)	
II	1060 (42.9)	243 (53.6)	817 (40.5)	
III	264 (10.7)	45 (9.9)	219 (10.9)	
Unknown	14	1	13	
Estrogen receptor status				0.715^a^
Positive	2256 (91.2)	412 (90.7)	1844 (91.3)	
Negative	218 (8.8)	42 (9.3)	176 (8.7)	
Unknown	9	0	9	
Progesterone receptor status				0.139^a^
Positive	1790 (72.6)	316 (69.8)	1474 (73.2)	
Negative	677 (27.4)	137 (30.2)	540 (26.8)	
Unknown	16	1	15	
HER2 receptor status				0.017^a,^*
Positive	208 (8.4)	51 (11.2)	157 (7.8)	
Negative	2260 (91.6)	403 (88.8)	1857 (92.2)	
Unknown	15	0	15	
Resection margin				0.167^a^
More than focally positive	123 (5.0)	30 (6.6)	93 (4.6)	
Focally positive	216 (8.7)	42 (9.3)	174 (8.6)	
Negative	2144 (86.3)	382 (84.1)	1762 (86.8)	

Data are expressed as *n* (%) unless otherwise specified. Missing cases are not included in the percentages

*HER2* human epidermal growth factor receptor 2, *MRI* magnetic resonance imaging, *N+* lymph node-positive, *N−* lymph node-negative (including isolated tumor cells)

^*^Denotes statistical significance at *p* < 0.05

^a^Chi-square test; missing values were not included

^b^Mann–Whitney U test; missing values were not included

^c^Data are median (25th and 75th percentiles). Tumor size was not known in two patients in the MRI group and one patient in the no MRI group

### Surgical Resection Margins

Overall, 123 (5.0%) women with invasive breast cancer had positive resection margins (more than focally involved margins). In absolute percentages, 6.6% (30/454) of the women with invasive breast cancer who underwent preoperative MRI had positive margins, compared with 4.6% (93/2029) in those who did not have preoperative MRI. In univariable analysis, preoperative MRI was not significantly associated with positive resection margins (odds ratio [OR] 1.47, 95% confidence interval [CI] 0.96–2.25) [Table 2]. However, after adjustment for possible confounders, the use of preoperative MRI was associated with a lower risk of positive resection margins, with an OR of 0.56 (95% CI 0.33–0.96). Confounders included breast density, mammographic abnormalities, tumor size, tumor histology, and lymph node status.

**Table 2:** Univariable and multivariable logistic regression analyses of associations with positive resection margins (more than focally [>4 mm] involved margins) in women with invasive breast cancer diagnosed after recall at screening mammography

	OR, univariable analysis (95% CI)	*p*-value	OR, multivariable analysis^a^ (95% CI)	*p*-value
Preoperative MRI				
No	1.0 (ref)	NA	1.0 (ref)	NA
Yes	1.47 (0.96–2.25)	0.074	0.56 (0.33–0.96)*	0.033*
Age, per year increase	1.01 (0.98–1.03)	0.657	NA	NA
Breast density at screening mammogram, %				
0–25	1.0 (ref)	NA	1.0 (ref)	NA
25–50	1.40 (0.89–2.23)	0.149	1.28 (0.79–2.08)	0.321
50–75	1.76 (0.98–3.16)	0.057	1.31 (0.68–2.53)	0.421
75–100	6.77 (2.71–16.90)*	<0.001*	3.61 (1.07–12.12)*	0.039*
Mammographic abnormality				
Mass	1.0 (ref)	NA	1.0 (ref)	NA
Microcalcifications	4.07 (2.55–6.49)*	<0.001*	4.45 (2.69–7.37)*	<0.001*
Mass with microcalcifications	1.25 (0.61–2.55)	0.537	1.33 (0.64–2.75)	0.446
Asymmetry	0.34 (0.05–2.51)	0.292	0.13 (0.01–1.43)	0.095
Architectural distortion	3.06 (1.75–5.35)*	<0.001*	1.85 (1.01–3.40)*	0.047*
Other	2.64 (0.61–11.54)	0.196	2.22 (0.40–12.33)	0.360
Tumor size, per mm increase	1.05 (1.04–1.07)*	<0.001*	1.05 (1.03–1.07)*	<0.001*
Tumor histology				
Ductal	1.0 (ref)	NA	1.0 (ref)	NA
Lobular	2.74 (1.75–4.30)*	<0.001*	2.86 (1.68–4.87)*	<0.001*
Mixed ductal-lobular	3.04 (1.47–6.30)*	0.003*	2.38 (1.07–5.31)*	0.034*
Other	0.65 (0.24–1.80)	0.409	0.74 (0.26–2.09)	0.571
Lymph node status				
N−	1.0 (ref)	NA	1.0 (ref)	NA
N+	1.96 (1.31–2.95)*	0.001*	1.53 (0.98–2.40)	0.060
Bloom and Richardson grade				
I	1.0 (ref)	NA	NA	NA
II	1.14 (0.77–1.68)	0.517	NA	NA
III	1.15 (0.63–2.10)	0.657	NA	NA
Estrogen receptor status				
Positive	1.0 (ref)	NA	NA	NA
Negative	0.91 (0.47–1.77)	0.784	NA	NA
Progesterone receptor status				
Positive	1.0 (ref)	NA	NA	NA
Negative	0.90 (0.60–1.34)	0.606	NA	NA
HER2 receptor status				
Positive	1.0 (ref)	NA	NA	NA
Negative	0.65 (0.37–1.13)	0.125	NA	NA

*CI* confidence interval, *HER2* human epidermal growth factor receptor 2, *MRI* magnetic resonance imaging, *NA* not applicable, *N+* lymph node-positive, *N−* lymph node-negative, *OR* odds ratio

Positive resection margins include specimens with more than focally involved (>4 mm) margins. Specimens with focally involved and negative margins were considered as tumor-negative margins

^*^Denotes statistical significance at *p* < 0.05. Missing values were imputed by multiple imputation

^a^Adjustment for variables associated with positive resection margins with *p* < 0.100 in univariable analysis

Irrespective of breast MRI use, the risk of having positive resection margins was higher in the presence of microcalcifications or architectural distortions compared with masses (OR 4.45, 95% CI 2.69–7.37, and OR 1.85, 95% CI 1.01–3.40, respectively). Furthermore, the risk of having positive resection margins was higher in breasts with a mammographic density of more than 75% compared with women with a breast density ≤25% (OR 3.61, 95% CI 1.07–12.12). The likelihood of having positive resection margins was almost three times higher in patients with invasive lobular cancer when compared with patients with invasive ductal cancer (OR 2.86, 95% CI 1.68–4.87) and the risk of having positive resection margins increased per millimeter increase in tumor size (OR 1.05, 95% CI 1.03–1.07).

## Discussion

Multivariable analysis showed that selective use of preoperative MRI in women with invasive breast cancer undergoing BCS after recall at screening mammography was associated with a lower risk of positive resection margins (more than focally [>4 mm] involved margins). Furthermore, we found that the presence of microcalcifications and architectural distortions, high (>75%) mammographic breast density, lobular histology, and increasing tumor size were independently associated with positive resection margins after BCS.

Surgical resection with tumor-free margins is one of the main challenges in BCS. According to the American Society for Radiation Oncology Consensus Guideline, no ink on tumor is the standard for an adequate margin in invasive breast cancer.^26^ However, in The Netherlands, re-excision or mastectomy is only recommended for more than focally positive resection margins, when >4 mm (one area or multiple areas) of invasive cancer and/or DCIS reach into the resection margins.^14^ Data from the Netherlands Cancer Registry showed that focally involved resection margins (≤4 mm involved) do not increase the risk of recurrence after local excision,^27^ which is why we adhered to the Dutch guidelines in this study. In total, only 5.0% of the women with invasive breast cancer in our study had more than focally involved resection margins, which is in line with a previous clinical audit performed in all hospitals in The Netherlands.^28^ Univariable analysis showed that the percentage of more than focally positive resection margins was higher in the MRI group than the no MRI group, although not significant. However, tumor characteristics of women who received preoperative breast MRI were less favorable than those without preoperative MRI. For example, women undergoing MRI had larger tumors and were more likely to have invasive lobular cancer, which were predefined criteria to perform MRI, as stated in the national guidelines. It is known that these factors increase the risk of positive resection margins,^1^,^29^–^31^ and this could have been the reason for performing MRI and thus be a source of selection bias, masking the real effect of preoperative MRI upon margin status. After adjustment for these and other potential confounders in a multivariable analysis, the use of preoperative breast MRI was associated with a lower risk of more than focally positive resection margins (adjusted OR 0.56, 95% CI 0.33–0.96; *p* = 0.033), indicating the added value of preoperative MRI in this screen-detected population.

To our knowledge, only one previous study investigated the association between preoperative MRI and margin involvement in women recalled at screening mammography.^1^ This study similarly concluded that preoperative MRI in patients with invasive breast cancer was associated with a lower risk of positive resection margins after BCS (adjusted OR 0.42; *p* = 0.015).^1^ However, Nederend et al.^1^ included women screened between 1997 and 2011, when the risk of positive resection margins was considerably higher compared with our study period (11.6% vs. 5%). Other studies that investigated the association between preoperative MRI and margin involvement mainly focused on symptomatic breast cancer patients and provided conflicting results.^4^,^5^,^31^–^36^ In line with the present findings, some studies showed that breast MRI use was associated with a reduced number of positive resection margins in patients with invasive breast cancer eligible for BCS.^1^,^4^,^31^,^34^ Lobbes et al.^31^ showed that the use of preoperative MRI was significantly associated with a lower risk of positive resection margins in patients with invasive breast cancer (adjusted OR 0.84; *p* = 0.015), which was mainly attributable to the effect observed in patients with invasive lobular carcinoma (adjusted OR 0.59; *p* < 0.001). Several other studies showed little or no effect,^5^,^33^,^35^,^36^ or even an unfavorable effect,^32^ of preoperative MRI on margin status. A plausible explanation of why additional information regarding MRI is not always translated into improved margin status is that MRI is usually performed in the prone position whereas surgery is performed in the supine position. Gombos et al.^37^ showed considerable change of the breast contour and tumor position between MRI performed in the supine and prone positions. They suggested that intraoperative supine breast MRI, in conjunction with standard prone breast MRI, may help in understanding the actual position of the breast tumors and thereby improve margin status. Moreover, the impact of MRI may depend on the experience of radiologists and surgeons, as well as on the multidisciplinary team communication. Differentiation as breast radiologist and surgeon should optimize mutual understanding and correct surgical planning.

It is often argued that preoperative MRI increases the likelihood of undergoing mastectomy. We observed that the fraction of patients who underwent preoperative MRI was larger in women who underwent mastectomy compared with women who underwent BCS. This is not unexpected since our study shows that the use of breast MRI increases with less favorable tumor characteristics and tumor size. Commonly, breast MRI is used to confirm the need for mastectomy or to enable the choice between primary surgical therapy or primary systemic therapy. However, as breast MRI in general shows more cancer than conventional imaging techniques, it is likely that the use of MRI in a number of patients has led to the performance of a mastectomy that would have been avoided without this evaluation. However, the low percentage of mastectomies (15.7%) shows that in women with screen-detected breast cancer this does not seem to be a major problem.

Irrespective of the use of preoperative MRI, the present study found that the presence of microcalcifications and architectural distortions, a high (>75%) mammographic breast density, lobular histology, and increasing tumor size were independently associated with positive resection margins. Similar to our findings, previous studies also reported lobular histology and large tumor size as risk factors for positive resection margins.^1^,^29^–^31^ Shin et al. also reported that a mammographic breast density of more than 75% was significantly associated with positive resection margins.^38^ High mammographic density makes localization and determination of the size of a tumor difficult^39^ and may thus explain why women with higher breast density are at higher risk of positive resection margins. The clinical impact of this result should be put into perspective, as <2% of the women in our study had a breast density of more than 75%. The higher risk for margin involvement in women with microcalcifications on mammography, observed in the present study, is also in line with previous studies.^1^,^29^,^38^,^40^ Microcalcifications are known to be associated with the presence of an extensive in situ component, the size of which is often underestimated by conventional imaging.^1^,^41^ We assume that microcalcifications on mammography indicate the presence of invasive cancer with DCIS, which could be considered an indication to perform MRI, as an extensive in situ component is known to be associated with involved resection margins after BCS.^1^,^30^ Architectural distortions on mammography were also previously found to be predictive for positive resection margins,^42^–^44^ which may be due to the fact that tumors characterized as architectural distortions can grow in a particularly infiltrative pattern, making it more difficult to determine resection margins.

The major strength of the present study was the large study population of women with screen-detected breast cancer. Our study is one of the few studies that evaluated the impact of preoperative MRI in screen-detected cancers, as most studies focused on clinically detected cancers. Moreover, our data provided insight into many variables, including imaging features, tumor characteristics, and surgical outcomes. However, extrapolation of our findings to other screening programs should be interpreted with caution as this study was performed in a Dutch screening population for which the design and work-up strategies differ from other countries. A second limitation is the non-randomized design of our study, which makes it difficult to exclude unrecognized differences between women who underwent preoperative MRI and those who did not. Finally, our sample size was too small for subgroup analyses on resection margins, such as for invasive ductal and lobular carcinomas. Furthermore, even though we included almost 2500 recalled women with invasive carcinoma, only 123 women (30 in the MRI group and 93 in no MRI group) had positive resection margins (>4 mm involved margins). This reduced the statistical power of our analyses.

## Conclusion

We found that selective use of preoperative MRI was associated with improved margin status after BCS in patients with screen-detected invasive breast cancer using multivariable analysis. Moreover, the presence of microcalcifications and architectural distortions, high (>75%) mammographic breast density, lobular histology, and increasing tumor size were independently associated with positive resection margins (>4 mm involved margins) after BCS. As most of these factors can be assessed preoperatively, they may improve surgical planning and reduce the risk of positive resection margins after BCS. Regarding our results, it can be argued to perform preoperative MRI in the high-risk settings described: microcalcifications, architectural distortions, high (>75%) breast density, and large tumors. Lobular histology is already being considered as an indication to perform preoperative MRI.
